# Peptide Receptor Targeting in Cancer: The Somatostatin Paradigm

**DOI:** 10.1155/2013/926295

**Published:** 2013-02-07

**Authors:** Federica Barbieri, Adriana Bajetto, Alessandra Pattarozzi, Monica Gatti, Roberto Würth, Stefano Thellung, Alessandro Corsaro, Valentina Villa, Mario Nizzari, Tullio Florio

**Affiliations:** Section of Pharmacology, Department of Internal Medicine and Center of Excellence for Biomedical Research (CEBR), University of Genova, Viale Benedetto XV, 2 16132 Genova, Italy

## Abstract

Peptide receptors involved in pathophysiological processes represent promising therapeutic targets. Neuropeptide somatostatin (SST) is produced by specialized cells in a large number of human organs and tissues. SST primarily acts as inhibitor of endocrine and exocrine secretion via the activation of five G-protein-coupled receptors, named sst1–5, while in central nervous system, SST acts as a neurotransmitter/neuromodulator, regulating locomotory and cognitive functions. Critical points of SST/SST receptor biology, such as signaling pathways of individual receptor subtypes, homo- and heterodimerization, trafficking, and cross-talk with growth factor receptors, have been extensively studied, although functions associated with several pathological conditions, including cancer, are still not completely unraveled. Importantly, SST exerts antiproliferative and antiangiogenic effects on cancer cells *in vitro*, and on experimental tumors *in vivo*. Moreover, SST agonists are clinically effective as antitumor agents for pituitary adenomas and gastro-pancreatic neuroendocrine tumors. However, SST receptors being expressed by tumor cells of various tumor histotypes, their pharmacological use is potentially extendible to other cancer types, although to date no significant results have been obtained. In this paper the most recent findings on the expression and functional roles of SST and SST receptors in tumor cells are discussed.

## 1. Somatostatin and Somatostatin Receptors: An Overview

Somatostatin (SST) is a cyclic neuropeptide containing a disulfide bond linking the cysteine residues at positions 3 and 14 (Cys_3_-Cys_14_). Native SST has two molecular forms, SST-28 and SST-14, consisting of 28 or 14 a.a., respectively, derived from proteolysis of a larger precursor molecule, pre-pro-SST. SST is ubiquitously expressed in humans, with high concentrations in brain, liver, lungs, pancreas, thyroid, gastrointestinal tract, and adrenal gland mainly acting as an inhibitor of exocrine and endocrine secretions on target organs. For example, SST suppresses GH, prolactin, and TSH production from pituitary gland, insulin, glucagon and exocrine secretions from pancreas, and several gastrointestinal peptides [1]. 

In the brain, SST acts as neuromodulator, with physiological effects on neuroendocrine, motor, and cognitive functions, and as neurotransmitter, exerting both stimulatory and inhibitory effects [2]. Moreover, the synthesis of many growth factors (insulin-like growth factor 1, IGF-1; epidermal growth factor, EGF; fibroblast growth factor, FGF; vascular-endothelial growth factor, VEGF) is also inhibited by SST [3, 4].

SST exerts its biologic effects by binding to five specific high-affinity receptors (sst1–5) on the cell surface [1, 5], belonging to the seven transmembrane, G protein-coupled receptor (GPCR) family. SST-14 and SST-28 bind all SST receptors with similar high affinity although SST-28 preferentially binds to sst5, while synthetic ligands display distinct SST-specific binding affinity (Table 1). SST receptors are encoded by different intronless genes (located on chromosomes 4, 17, 22, 20, and 16), except for sst2 existing in two splice variants which differ in the length of the carboxyl terminus: a long variant of 369-a.a. (sst2A) and a shorter one of 346-a.a. (sst2B) [1, 5]. Recently, novel truncated functional isoforms of sst5 have been identified [6, 7] in humans and mice.

On the basis of sequence identity and pharmacological features, two subfamilies of SST receptors were described: the first class, including sst2, sst3, and sst5, binds synthetic SST analogs such as octreotide and lanreotide, whereas components of the second group, sst1 and sst4, do not interact with these agonists [8]. This has relevant implications in SST-based diagnostic imaging and pharmacological approaches.

Cortistatin (CST), a novel neuropeptide of the SST family, was cloned in 1997 [9]. Two different mature CST peptides of 14 and 29 a.a., derived from the processing of a human precursor (pre-pro-CST), were identified (CST-14 and CST-29, respectively), CST binds all five SST receptors with similar affinity (Table 1) and CST-14 and shares 11 of the 14 amino acids of SST-14, as well as many physiological effects, such as neuroendocrine activity (inhibition of GH release), regulation of sleep rhythms, memory process, locomotion, and modulation of the immune system [10, 11]. More recently, two putative CST receptors, that do not bind SST were also identified: MrgX2 (MAS-related gene X2) receptor [12] and GHS-R1a (growth hormone secretagogue receptor 1a) [13], whose biological function is still not completely characterized. 

## 2. Somatostatin Receptor Expression and Functions in Normal Tissues 

SST receptors are differentially expressed in discrete or overlapping distribution in multiple target organs, such as central nervous and immune systems, pituitary, thyroid, and adrenal glands, pancreas, gut, and kidney. This complex pattern of SST receptor expression includes coexpression of multiple subtypes in a tissue-specific pattern and distinct physiological roles [14].

SST receptors are widely expressed throughout the brain: in particular, sst1 and sst2 have a diffuse localization, whereas sst4 and sst5 show a more confined expression in hippocampus and hypothalamus, respectively [15]. Moreover, in different brain regions, sst1 is also localized presynaptically, controlling SST release from somatostatinergic neurons [16]. Starting from animal studies showing a positive modulation of exogenous administered SST on memory retention [17, 18], and the reduction of SST expression during aging [19], recent evidence proposed a key role of SST neuron dysfunction in Alzheimer's disease and other brain disorders associated with cognitive impairment (for a review see [2]). Interestingly, SST receptors also affect glial function, for example controlling IL-6 secretion from astrocytes [20]. SST receptors are also localized in the peripheral nervous system, in proximity of pain terminals, where they were reported to play a role in nociception [21].

At pituitary gland level, sst1 and sst5 mainly control GH and prolactin secretion while sst2 is involved in the release of GH, TSH, and ACTH [1]. 

In the immune system, SST acts as autocrine/paracrine factor within the complex cellular structure of lymphoid organs. The presence of SST and its receptors was established in cells mediating inflammation and immune response such as B and T lymphocytes and in monocytes, but not granulocytes and the specific expression of sst3 on peripheral human T lymphocytes seems to vary with species and with the origin of T cells [22]. sst1, sst2, and sst3 were identified in thymus with a higher expression of sst2 in immature thymocytes (CD2^+^/CD3^−^) and sst3 mainly in more mature cells (CD3^+^). Immune response and neuroendocrine regulation occurs at multiple levels and, among different immunoregulatory peptides, SST is involved in the control of cell growth and migration [23]. CST also is involved in the modulation of inflammatory response, acting as endogenous anti-inflammatory neuropeptide. CST is produced by T cells, macrophages and monocytes in response to inflammation, injuries, or antigen stimulation and, through the binding to both sst1–5 and ghrelin receptors expressed on immunocompetent cells, inhibits the release of inflammatory factors, chemokines, T cell proliferation, and T helper cell response and stimulates IL-10 production and regulatory T cells [24].

In the gastrointestinal tract, the mucosal delta-cells are one of the major sources of SST in the gut influencing motility, secretion and absorption [25]. At the pancreatic level, both sst1 and sst5 are highly expressed in insulin-releasing beta-cell, sst5 in the SST-releasing delta-cells and sst2 mainly in glucagon secreting alpha-cells. On the contrary, sst3 and sst4 are poorly expressed. In intestinal cells, sst5 controls the release of glucagon-like peptide-1 (GLP-1).

## 3. Somatostatin Receptor Signal Transduction, Homo- and Heterodimerization, and Trafficking

The different physiological effects of SST in different tissues are mainly ascribed to the specific characteristics of the cell types that express the SST receptors, resulting in the activation of distinct signal transduction pathways. In detail, the inhibition of adenylyl cyclase activity and the reduction of intracellular Ca^2+^ levels through the coordinated activities on different K^+^ and Ca^2+^ channels, are responsible of the antisecretory effects of SST, while the activation of phosphotyrosine phosphatases (PTPs), and in particular of three enzymes of this family: Src-homology phosphatase type 1 (SHP-1) and type 2 (SHP-2), and density-enhanced phosphatase 1 (DEP-1)/PTP*η*, and the modulation of mitogen-activated protein kinase (MAPK) activity, are mainly responsible of SST antiproliferative effects [26–43]. Both adenylyl cyclase inhibition and PTP activation are induced by all SST receptor subtypes, while MAPK activity is increased by sst4, decreased by sst3 and 5, and modulated in both directions by sst1 and 2. Moreover, SST/receptor interaction acts on K^+^ and voltage-gated Ca^2+^ channels, NA^+^/K^+^ exchanger, cyclooxygenase-2 and (sst2 and 5), and phospholipase A2 (sst1 and 2) activities [44, 45].

**Figure 1:**
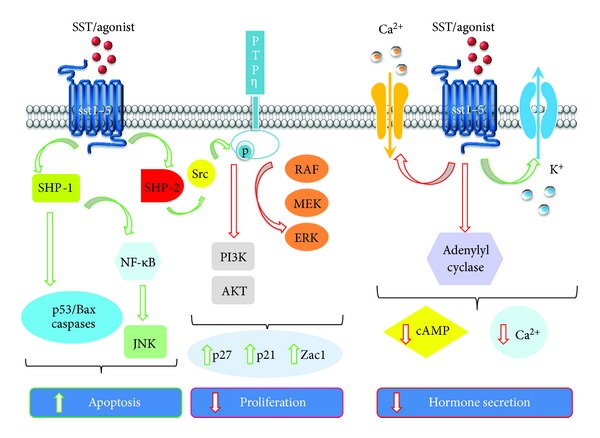
Schematic representation of the intracellular signaling pathways modulated by somatostatin receptors. Antiproliferative effects of somatostatin (SST) and its analogs; SST and analogs binding to SST receptors activate different phosphotyrosine phosphatases (PTPs) SHP-1 and SHP-2 and PTP*η*. Activated SHP-1 triggers intracellular proapoptoptic signals involving the induction of caspase activation and p53/Bax. SHP-1 also cause apoptosis by activation of the transcription factor NF-*κ*B leading to the inhibition of the MAP kinase JNK anti-apoptotic effects. SHP-2 activates Src that directly interacts with PTP*η* inducing its phosphorylation in tyrosine and activation. PTP*η* dephosphorylates intracellular effectors involved in the control of cell cycle progression, such as the ERK and the PI3K/Akt pathways, causing the upregulation of the cyclin kinase inhibitors p21^cip1/waf1  ^ and p27^kip1^ and the tumor suppressor gene Zac1. As a result, cells accumulate in G1 phase without entering S-phase and cell proliferation is blocked. Antisecretory effects of SST and its analogs; SST inhibits the secretion/synthesis of many hormones through the inhibition of voltage-dependent Ca^2+^ channels and activation of K^+^ channels, decreasing intracellular Ca^2+^ concentration, and inhibition of adenylyl cyclase, lowering intracellular cAMP levels. Activated pathway: green arrows; inhibited pathway: red arrows.

An overview of the signaling pathways activated by SST receptors is summarized in Figure 1 and Table 2.

The role of specific SST receptor subtypes in SST antiproliferative effects have been identified, since sst1, 2, 4, and 5 are mainly involved in the arrest of cell cycle progression, while sst2 and 3 are also able to activate proapoptotic pathways, as well as anti-angiogenic signals, an indirect control of tumor growth [44, 45].

**Figure 2:**
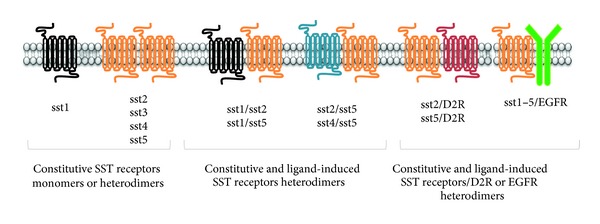
Dimerization pattern of somatostatin receptors. Each SST receptor subtype show a different constitutive tendency to dimerize. sst1 exists as a monomer while sst2, 3, 4 and 5 homo-dimerization occurs both constitutively or after somatostatin binding. SST receptors may form heterodimers with other member of SST receptor family, either in resting conditions or upon ligand binding. SST receptors also dimerize with receptors of other GPCR families, (e.g., D2R) or with other receptor families, such as tyrosine kinase receptors (i.e., EGFR), originating heterodimers.

Homo- and/or heterodimerization of all GPCR family, including SST receptors, play(s) a fundamental role in ligand binding, receptor expression, trafficking and desensitization, and signal transduction [46] due to distinct biological features of the native monomeric isoforms. The functional interactions among individual human SST receptors have been extensively investigated [47] as well as the dimerization with different components of SST receptor family or with other GPCRs [48, 49] (see Figure 2). Dimerization was shown to be either constitutive or ligand-dependent and, despite high structural homology, SST receptor subtypes show marked dimerization heterogeneity and differences between different species. sst1 is the only SST receptor existing as monomer, regardless agonist binding [50]. In basal conditions, sst2 and sst3 exist as homodimers but, in response to agonists, dissociate to monomers that are rapidly internalized for sst2, while monomers of sst3 are retained in membrane [51]. On the contrary, basal sst4 homodimers are enhanced after ligand treatment [52]. sst5 is monomeric and displays dimerization upon agonist binding in a concentration-dependent manner. 

SST receptor subtypes, coexpressed in the same cells, can also form heteromeric complexes within SST receptors, or heterodimerize with members of different GPCR family (i.e., dopamine, opioid, or adrenergic receptors). sst1/sst5, sst2/sst3, and sst4/sst5 heterodimerizations have been described, although these studies mainly refer to heterologous cell models (CHO-K1 or HEK-293) transfected with specific SST receptor subtypes [49, 51].

Among the interactions of SST receptors with components of other GPCR families, those with dopamine 2 receptor (D2R) have been widely investigated for their potential therapeutic implications [53]. In fact, SST receptors and D2R are frequently coexpressed in endocrine and pituitary tumors [54] and both receptor activations lead to inhibition of hormonal secretion and cell proliferation. *In vitro* receptor-transfected models showed that D2R preferentially forms heteromeric complexes with sst2 and sst5 [48, 49]. These results were confirmed in non-endocrine tumor cells, natively expressing SST receptors and D2R. It was shown that these receptors can interact in the absence of agonists [55] and the treatment with the chimeric compound targeting both receptor types (BIM-23A760) significantly increased the sst5/D2R and sst2/D2R dimers. 

A cross-talk between SST receptors and growth factor tyrosine kinase receptors (RTKs) (e.g., epidermal growth factor receptor, EGFR) has been recently described [4], resulting in sst1 or sst1/5 dimers binding to EGFR to negatively regulate EGF-mediated effects [3, 56]. The relevance of heterodimerization between SST receptors and EGFR is mainly due to the involvement of this RTK in mitogenic signaling and oncogenic transformation, as SST receptors may competitively bind to EGFR, interfering with its phosphorylation and activation of signaling pathways responsible of cancer development [4]. In fact, it was described that in pancreatic cancer cells activation of SST receptors results in a PTP-dependent, pertussis toxin-sensitive dephosphorylation of EGFR [57].

As previously discussed, SST receptor trafficking (endocytosis, recycling or degradation) after agonist binding is well documented [58]. On the contrary, less is known about functional SST receptor travelling along the secretory pathway to the plasma membrane. Upon agonist activation, SST receptors undergo phosphorylation through G-protein-coupled receptor kinase (GRK) and bind *β*-arrestin-1 and -2 that trigger the desensitization process by blocking coupling between the receptor and G proteins [59]. Phosphorylated receptors are then internalized into clathrin-coated vesicles and directed to endosomes, where they are dephosphorylated by specific serine/threonine phosphatases. Dephosphorylated SST receptor complexes can be recycled/resensitized on cell membrane [60] or degraded in different intracellular compartments.

Individual SST receptors have different internalization rates (internalization is higher for sst2, 3 and 5 than for sst1 and 4) [61], since their interaction with *β*-arrestins results in receptor/arrestin complexes with lower or higher stability. This specific feature influences SST receptor fate: sst2 and 5 are faster recycled while sst3 commonly undergoes degradation [61, 62]. In addition, diverse SST analogs may affect SST receptor trafficking in different ways, as demonstrated by the SST agonist pasireotide that either causes sst2 membrane recycling [63] or does not affect its internalization [64], whereas octreotide mainly induces sst2 internalization.

Importantly, starting from this evidence, a very recent study [65], evaluating the ability of different SST analogs to induce SST receptors internalization, was able to classify them as agonists, partial agonists, or antagonists toward sst2 3 and 5, through the determination of receptor biological responses instead of evaluating only binding affinity. Unexpectedly, in this experimental model, pasireotide (showing high affinity binding for sst1, 2, 3, and 5) was able to activate sst3 and 5 but acted as partial agonist at the sst2 [65]. Octreotide (able to bind with high affinity sst2 and 5) exhibited agonistic properties for sst2 but failed to activate sst5. Finally, the recently introduced SST analogs, somatoprim (that binds with high affinity to sst2, 4 and 5) was a full agonist for both sst2 and sst5 [65]. Thus, it was proposed that a reevaluation of specific effects of SST agonists toward each subtype should be performed, although homo- and heterodimerization could further complicate the analysis.

## 4. Somatostatin Analogs 

The biological effects induced after SST receptor activation allowed their identification as relevant drug targets. However, the main limit in using native SST for *in vivo* therapy is the very short half-life of this peptide. Thus, to prolong the biological activity of SST many analogs have been synthesized, showing a prolonged persistence in the organism and often endowed of increased potency. Among them, the first octapeptide developed was octreotide, which reaches a half-life of 90–120 min after subcutaneous administration, followed by lanreotide, and vapreotide, also being cyclooctapeptide SST analogs [1]. 

**Figure 3:**
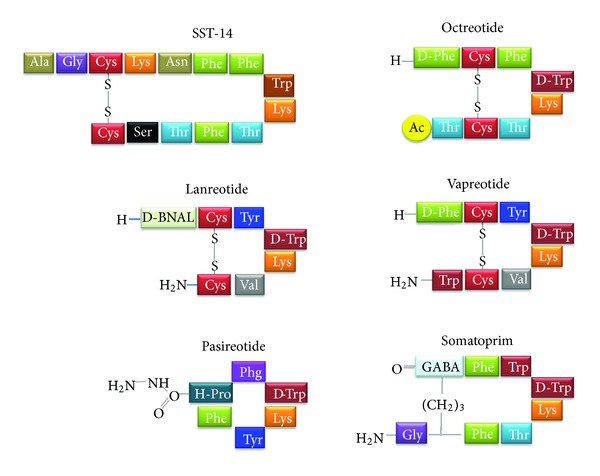
Amino acid composition of somatostatin 14 (SST-14) and of the main synthetic analogs (octreotide, lanreotide, vapreotide, pasireotide, and somatoprim).

Primary structures of native SST-14 and of the most relevant synthetic analogs are reported in Figure 3, and the calculated IC_50_ toward individual SST receptor subtypes of all these compounds is reported in Table 1. 

A common pattern of SST receptor binding ability is shared by these analogs: high affinity for sst2 and sst5, medium/low affinity for sst3, and lack of binding to sst1 and sst4 [1].

Octreotide and lanreotide are currently approved for the treatment of GH-secreting pituitary adenomas. More recently, improved formulations of the above molecules were introduced in clinical use, such as octreotide long-acting-release (LAR), lanreotide-sustained-release and lanreotide autogel, that improved the pharmacokinetic parameters allowing a single administration every 14 or 28 days [66, 67]. 

The possibility of a selective modulation of a single SST receptor subtype, to inhibit specific biological functions, represents an attractive pharmacological feature to obtain therapeutic specificity. A number of monospecific SST receptor subtype analogs were developed being either peptidomimetic compounds [68] or peptides, such as sst1-specific (BIM-23745 and BIM-23926) sst2-selective (BIM-23197 and BIM-23120), and sst5-specific (BIM-23268 and BIM-23206). These compounds were reported to mediate both antisecretory and antiproliferative activity in experimental models [69].

More recently, based on molecular investigations of cell SST receptor expression profile showing in most cases coexpression of multiple receptors, it was developed the concept that a better pharmacological response could be obtained through the simultaneous activation of all the receptors in a given tissue. This assumption led to the identification of novel compounds able to bind simultaneously two SST receptors with higher affinity than reference drugs (octreotide and lanreotide). Among them the bispecific molecules BIM-23704 (sst1 and 2) and BIM-23244 (sst2 and 5) were used in several preclinical studies [69, 70]. Subsequently, multireceptor binding analogs (i.e., pasireotide (SOM230), somatoprim, KE108, and BIM-23A779) were developed to overcome octreotide resistance in acromegaly and carcinoid tumors and to increase sensitivity in pathologies of tissues expressing SST receptors other than sst2 [71–73]. In this group pasireotide is one of the newest multi-receptor ligand analog, exhibiting a prolonged half-life, and high affinity for sst1, 2, 3 and 5, with 30-to-40-fold higher affinity for sst1 and 2 than octreotide or lanreotide [73]. Pasireotide represents a promising candidate for octreotide resistant or lanreotide low-responsive tumors [75]. Therefore, at the end of a 12-months phase III study, pasireotide has been approved for Cushing's disease [76], and it is currently in phase III clinical trials for acromegalic patients and phase II trials for neuroendocrine tumors (NETs). 

Other pan-SST receptor agonists are currently under development: the hepta-backbone cyclic peptide somatoprim (DG3173), which is currently under clinical and preclinical evaluation, KE108 with a reduced size and stabilized structure, and BIM-23A779 (see Table 1) [71, 77]. Somatoprim exhibits a unique binding profile in that binds with high affinity to sst2, 4 and 5 but not sst1 and 3 [78].

Several potent and selective sst4 binding peptides and nonpeptide compounds have been synthesized. Among them TT-232, a hepta-peptide sst4 and sst1 agonist, and J-2156, sst4-selective compound, have shown anti-inflammatory, antinociceptive and antitumor effects [79–81].

Although less-represented sst3 agonists and antagonists have been also synthesized: BIM-23056 is a sst3 agonist with antagonist activity for sst5, analogs with replacement of the tryptophan residue by one of the two enantiomers of 3-(3′-quinolyl)-alanine binds sst1 and 3 [82], while BN81658, is a selective sst3 antagonist [83]. 

Chimeric compounds able to bind both SST and dopamine (DA) receptors were synthesized, on the basis of simultaneous presence and heterodimerization of these receptors on the same normal and tumor cells in pituitary gland and gastrointestinal tract, and on the clinical evidence that the cotreatment with octreotide and the DA agonist bromocriptine inhibits GH secretion in acromegalic patients more efficiently than the single agents [54, 84].

The compound BIM-23A387, selective for sst2/D2R, showed the *in vitro* ability to strongly block GH and PRL release in human pituitary cells as compared to sst2- and D2R-monospecific analogs [85]. Hormonal hypersecretion is efficiently controlled also by sst2/D2R-high-affinity/sst5-low-affinity chimeric molecule BIM-23A760 [53, 77]. These molecules tested in a non-small lung cancer cell line, and in a prostate cancer cell line showed a greater antiproliferative effect than subtype specific SST and DA agonists, alone or in combination [55, 86], and in the control of cell growth from primary cultures of human nonfunctioning pituitary adenomas [53]. However, recently, a phase II trial with BIM-23A760 in acromegaly was stopped due an unexpected time-dependent accumulation of metabolites that impaired the activity of the native molecule [87].

The above data highlight the great efforts and developments achieved in SST receptor-targeted therapy. However further studies are needed to delve deeper the pathophysiological role of SST receptor homo- and heterodimerization, trafficking, and their cell and tissue specificity as factors that may influence SST analogs clinical responses to develop more efficacious and selective drugs.

## 5. Radioactive Somatostatin Analogs for Imaging and Therapy

Radiolabeled peptides have acquired increasing interest in nuclear medicine for tumor diagnosis, staging, therapy (peptide receptor radionuclide therapy, PRRT) and follow-up. In particular, the use of SST analogs as radiolabeled peptides is a powerful diagnostic tools for *in vivo* tumor imaging, since, after intravenous injection they accumulate in SST receptor-expressing tumors [88]. In parallel, due to the cytotoxicity of radiolabeled SST analogs, an emerging and effective treatment strategy is SST-based PRRT.

Diagnostic SST radioisotopes allow the localization of SST receptor expressing tissues and identify patients for subsequent radionuclide therapy. The structure of these labeled peptides includes a chelator (e.g., diethylenetriamine-pentaacetic acid, DTPA, or 1,4,7,10-tetraaza-cyclododecane-1,4,7,10-tetraacetic acid, DOTA) that forms a stable complex with the radiometal-conjugated SST analogs. These three elements (chelator, radiometal, and SST analog) strongly influence the efficacy of imaging and therapy, affecting receptor binding affinity, internalization rate, and *in vivo* label stability; thus, the development of novel radiopeptides focused on the improvement of these properties to obtain suitable compounds. 

 [^111^In]-DTPA-octreotide retains high affinity for sst2 and has been used for scintigraphic imaging for primary and metastatic neuroendocrine tumors (NETs), improving the sensitivity of standard diagnostic modalities [89]. It was further optimized by molecules as DOTA-d-Phe1-Tyr3-octreotide (DOTATOC), DOTA-d-Phe1-Tyr3-octreotate (DOTATATE), characterized by higher sst2 affinity, and DOTA-1-NaI Tyr3-octreotide (DOTANOC) that also binds sst3 and sst5. More recently, the availability of positron emitters ([^68^Ga] and [^18^F]) labeled analog (DOTATOC) allowed its application to positron emission tomography (PET) imaging [90], overcoming weakness of *γ*-radiation emitters for SPECT as of [^111^In]-DTPAOC (Octreoscan) in the detection of metastatic NETs [91, 92].

In endocrine tumors, PRRT with SST analogs was initially performed using [^111^In]-coupled peptides, then replaced by DOTA stably labeled with [^68^Ga], [^90^Y], or [^177^Lu]. These compounds were well tolerated and efficacious in controlling symptoms and prolonged patient survival [93]. In a large study on 504 NET patients treated with the radiolabeled SST analog [^177^Lu-DOTA,Tyr_3_]-octreotate [90], an overall survival benefit was evident when the outcome was compared with the historical data of the group. Imhof et al. [94] reported a phase II study on a large series of patients with metastasized neuroendocrine cancers treated with repeated cycles [^90^Y-DOTA]-TOC reporting that response is associated with longer survival. 

Currently, single radioisotope therapy is the standard PPRT practice, however preclinical study in rats bearing pancreatic cancer, showed that the association of [^77^Lu]- and [^90^Y]-SST analogs exerts higher antitumor efficacy than single radionuclides [93]. Another study, evaluating the effectiveness of SST-based radiopeptide therapy using single radioisotope *versus* a combination of compounds in patients with NET metastatic cancers showed that the combination of [^90^Y-DOTA]-TOC + [^177^Lu-DOTA]-TOC radiopeptides improved overall survival as compared with [^90^Y-DOTA]-TOC alone, while comparable toxicity was observed in the two groups [95]. 

Radiopeptides used in clinics are SST agonists, mainly octreotide, on the basis of their high-affinity binding and the induction of internalization of the ligand-receptor complex that leads to intracellular accumulation of the radioligand [96]. However, preclinical *in vivo* studies showed that sst2- and sst3-expressing tumors have higher uptake of radiolabeled SST antagonist, as demonstrated by the evaluation of the labeled antagonist [^111^In]-DOTA-sst3-ODN-8 in a mouse model bearing sst3 expressing tumor and by *in vitro* experiments with [^111^In]-labeled sst2 antagonist DOTA-sst2-ANT ([^111^In]-DOTA-[4-NO_2_-Phec(DCys-Tyr-DTrp-Lys-Thr-Cys)-DTyr-NH_2_ [97, 98]. Recently, bicyclic SST-based analogs have been proposed as potential radiopeptides for SPECT/PET on NETs [99].

Other radioantagonists were developed for PET in sst2-positive tumors as [^64^Cu] and [^68^Ga] antagonists coupled with different chelators [4,11-bis(carboxymethyl)-1,4,8,11-tetraazabicyclo [6.6.2]hexadecane (CB-TE2A), 1,4,7-triazacyclononane,1-glutaric acid-4,7-acetic acid (NODAGA), and DOTA] showing a higher uptake and image contrast on PET scans [99].

A recent pilot study is evaluating the clinical feasibility of imaging with the SST receptor antagonist [^111^In]-DOTA-pNO_2_-Phe-c(DCys-Tyr-DTrp-Lys-Thr-Cys)DTyrNH_2_ ([^111^In]-DOTA-BASS) in patients with NETs [100].

Beside NETs, SST-based tracers ([^111^In]-pentetreotide) may represent a diagnostic tool in tumors expressing sst2, 3, and 5, such as meningiomas, gliomas, Hodgkin and non-Hodgkin lymphomas, and metastases from breast cancer [101]. In other lesions (pheochromocytomas and medullary thyroid carcinomas) a definite usefulness of radiolabeled SST analogs is still not proven, mainly due to their limited detection rate [102, 103]. Similarly, conflicting results using [^111^In]-DTPA-octreotide in pituitary adenomas were reported [103]. Few case-report studies demonstrated the efficacy and safety of radiolabeled SST-analogs in pituitary metastasis in a patient with NET [104] and in a case of relapsed prolactin-secreting giant pituitary adenoma [105]. 

Thus SST receptor targeting with radiolabeled analogs is an important development, particularly in NET imaging and therapy, and radiopeptide translational research may support innovative therapeutic modalities [106, 107].

## 6. Somatostatin Receptor Signaling in Tumor Cells

SST receptors are commonly expressed in tumors including both endocrine (pituitary adenoma, neuroendocrine and gastropancreatic neoplasms, thyroid, adrenal, and small cell lung carcinomas) and nonendocrine (gliomas, meningiomas, breast, and ovarian cancers, osteosarcomas) histotypes. Generally, sst2 is the subtype most widely expressed and sst3 and sst4 the less expressed in human cancers. Although in most cases all subtypes can be identified in tumor cells a tumor-specific pattern of expression can be identified in some cases. For example, sst2 and sst5 are the receptors more frequently detected in GH-secreting pituitary adenomas, while sst3 is frequently expressed, altogether with sst2 and 5, in nonfunctioning pituitary adenomas [108].

SST ability of inhibiting tumor growth and metastatic spread involves SST receptor activation located on both cancer and microenvironment cells, particularly endothelial cells of tumor vessels responsible of the neovascularization of the tumor.

The antitumor activity of SST and its analogs is mediated by multiple SST receptors, through direct antiproliferative (inhibition of mitogenic stimuli of growth factors, arrest of cell cycle) and proapoptotic signals and, indirectly, inhibiting the secretion of proliferative and angiogenic growth factors and hormones or affecting neoangiogenesis at endothelial cell level [44]. 

SST controls cancer cell proliferation via the interference with different signaling pathways (PTPs, JAK2, Ras/ERK, and Pi3K/Akt) resulting in cytostatic effects mediated by the induction of involving the induction of cell cycle inhibitors p27 or p21, or tumor suppressors, like Zac1 [33, 109–112]. 

Studies using cells transfected with individual SST receptor subtypes, or using selective agonists in cell natively expressing multiple SST receptors, showed that all 5 receptor activations induce PTP activity [27, 37, 40, 57, 69, 113, 114]. To date, a subset of PTPs, namely, the cytosolic SHP1, SHP2, and the transmembrane PTP*η*, are considered the main effectors of the antiproliferative activity of SST. These enzymes, upon activation, through the modulation of several transduction mechanisms, target tyrosine residues of specific substrates regulating proliferative pathways (ERK1/2 PI3 K/AKT, NO/cGMP, etc.) [110, 115–119].

Arrest of cell proliferation, related to SST receptor activation of SHP1, was reported in different tumor cell lines derived from pituitary adenomas (GH3) and breast (MCF-7), pancreatic (MIA-PaCa, PANC-2, PC-1, PC-3, AR4-2J), and thyroid medullary (TT) carcinomas [110, 114, 120–124]. In co-immunoprecipitation experiments, sst2 and SHP-1 were identified in a multiprotein complex regulated by Gi3*α* [125]. Octreotide activation of sst2 caused the activation of SHP-1 that rapidly dissociates from the receptor, binds to insulin receptor dephosphorylating the receptor itself and its substrates (i.e., IRS-1 and Shc) and leading to a negative modulation of insulin mitogenic signalling [126]. sst2 antiproliferative effects, characterized by the inhibition of the entry in the S phase and the accumulation of the cells in G1, due to p27^kip1^ overexpression that sequesters cdk2 from cyclin E and induces accumulation of hypophosphorylated retinoblastoma gene product [112]. SST and analogs activation of SHP1 resulted in tyrosine phosphorylation of the PTP, mediated by the cytosolic kinase JAK2 whose activity was identified as an absolute requirement for both sst2-mediated activation of SHP1 and the inhibition of cell proliferation [127]. In resting conditions, sst2, JAK2, and SHP1 form a unique signalling complex, but upon SST binding JAK2 dissociates and phosphorylates and activates SHP1 [128]. Moreover, SHP2 and cytosolic tyrosine kinases (c-Src) were also detected in the sst2-associated multieffector complex [128]. Thus, it was proposed a molecular model in which the cytostatic effects of SST, via sst2, are the results of the sequential activation of kinases and phosphatases, with SHP2 phosphorylation by c-Src responsible of SHP1 binding to the receptor and activation (for review see [129]).

SHP2 was also involved in SST effects on cell cycle mediated by sst1 activation [28, 33, 40]. In particular, in CHO-K1 cells, solely expressing sst1, SST induced cytostatic effects through a rapid activation of SHP2 and c-Src [33]. SHP2 is expressed in several SST-responsive tumors including gliomas and neuroblastomas as well as in thyroid cells. Its activation by SST receptors causes cell cycle arrest via the inactivation of the tyrosine kinase receptors for EGF, platelet derived growth factor (PDGF) and insulin [57, 109, 130] and the subsequent inhibition of the growth factor-dependent activation of ras/Raf/ERK1/2 pathway [131]. However, kinetics studies showed that sst1 activation in CHO-K1 cells also caused a delayed and long-lasting PTP activity, besides SHP2 whose activation was, on the other hand, rapid and transient [28]. In rat thyroid cells, this activity was associated to the PTP*η* (also called DEP-1 in humans) a membrane bound receptor-like PTP whose activation causes dephosphorylation of ERK1/2. The reduced activity of MAP kinase resulted in a low level of phosphorylation of the CDKI p27^kip1^ that, in the unphosphorylated form, cannot be ubiquitinated and degraded by the proteasome [38, 109, 132]. These results were confirmed studying glioma cells, in which SST-activated PTP*η* was able bind active ERK1/2 causing its dephosphorylation/inactivation [133] and the consequent upregulation of p27^kip1^ [134]. Importantly, in glioma cell lines and primary cultures from human glioblastomas, the cytostatic activity of SST was dependent on the expression and activation of PTP*η*. However, while SHP2 is widely expressed in gliomas, PTP*η* expression is rather inconstant (it was detected in about 1/3 of the human glioblastomas analysed, [133]), and the possibility to activate this PTP by SST agonists was proposed to represent a potential molecular determinant to obtain antiproliferative responses in glioblastomas [133].

Thus, from these studies it was proposed that two classes of PTPs are activated in response to SST: SHP1 and SHP2 that act mainly on activated tyrosine kinase receptors and PTP*η* that mainly acts down-stream the proliferative signalling dephosphorylating ERK1/2. However, in the same way described for sst2 and SHP1 (see before), the activation of PTP*η* by sst1 requires the constitution of a multieffector complex, composed of both kinases and PTPs. In these studies using either cells natively expressing sst1 (C6 glioma cells) or CHO-K1 cells transfected with this receptor, a large multimeric protein complex occurred in proximity of sst1 in resting conditions that, besides the receptor, was composed of the trimeric G-protein, Jak2, SHP2, c-Src, and PTP*η* [135]. Upon SST binding via G protein activation Jak2 becomes active and phosphorylated SHP2. Upon phosphorylation, SHP2 is also activated, dissociates from the receptor and dephosphorylates the inhibitory tyrosine on c-Src C-terminus. Active c-Src, in turn, phosphorylates PTP*η* causing the sustained activity of this PTP to inactivate ERK1/2 [135]. 

The identification in different cell models of similar multieffector cascades activated by different SST receptors (sst1 and sst2) that, through the interplay of different kinases (Jak2, c-Src) and PTPs (SHP2), leads to the activation of an effector PTP (SHP-1 or PTP*η*), allowed the definition of a common modular transducing mechanism by which cytostatic effects are induced by SST [129].

Another direct mechanism by which SST controls cell growth is the induction of pro-apoptotic pathways mainly mediated by sst2 and sst3 and involving SHP-1. Apoptosis may occur via extrinsic (activated by the receptors of the TNF*α*-related apoptosis-inducing ligand), and Fas ligand or intrinsic (mitochondrial) pathways. sst2 inhibits the PI3K/AKT cascade, the anti-apoptotic protein Bcl-2 and NF-*κ*B transcription factor, while sst3 induces apoptosis via the activation Bax [136–139]. Table 2 summarizes the different pathways of SSTR subtype activation.

Indirect antiproliferative effects of SST involve the inhibition of the GH/IGF-I axis through central (sst2, sst5) and peripheral (sst2, sst3) mechanisms, which via a PTP, dephosphorylate STAT5b and inhibit expression of the hepatic IGF-I [140].

SST receptor expression was observed in peritumoral vessels, mainly in endothelial cells. In particular, sst2 has been found to be uniquely upregulated during the angiogenic switch, from quiescent to proliferative endothelium [141]. Indeed, SST is a powerful inhibitor of neovascularization in several experimental models and, consequently, the inhibition of tumor angiogenesis is considered one of the mechanisms mediating SST antineoplastic effects. Antiangiogenic properties of SST were identified to be mediated by sst2 and sst3 activation that results in the blockade of proliferation and migration of endothelial cells and of monocyte activation [142], and the inhibition of the release of proangiogenic factors such as VEGF, PDGF, IGF-1, and bFGF [141]. The effects on endothelial cells were mainly mediated by the inhibition of eNOS and ERK1/2 activities [143]. Inhibition of NO production by both eNOS and nNOS was also described after sst1–3, but not sst4, activation [115] via the interference with different molecular mechanisms involving both classical (PLC activity and PI3k/Akt) and novel (ceramide synthesis) pathways [144].Conversely, nNOS was activated in rat retina by sst2 agonists via an SHP1-dependent mechanism [145].

Tumor cell invasiveness, a feature characterizing the aggressiveness of malignant tumors, also represents a SST receptor target via the inhibition of the PI3K/Akt pathway and the modulation of proteins responsible of actin filament assembly (namely, Rac and Rho) [146]. 

## 7. Somatostatin and Somatostatin Receptors in Neuroendocrine Tumors

SST receptors are highly expressed in neuroendocrine tumors (NET, including pituitary adenomas, endocrine pancreatic tumors, gastrointestinal and lung carcinoids, small cell carcinomas, thyroid medullary cancer, and others). Pituitary adenomas and gastroenteropancreatic (GEP) NETs represent the major tumor targets for SST analogs presently used in clinical practice.

Pituitary tumors are generally benign slow-growing neoplasms, and different adenomas show a typical pattern of SST receptor expression [74] according to the secreting cells from which they originate: GH-secreting pituitary adenomas mostly express sst2 and sst5 [147], ACTH-secreting lesions predominantly coexpress sst5 and sst2 [148] while in prolactinomas sst1 and sst5 are the predominant receptors [149]. In clinically nonfunctioning pituitary adenomas sst3 is highly expressed, followed by sst2 and, at low level, sst5 [53, 150], while, in TSH-secreting tumors, sst2 is mainly coexpressed with sst3 and sst5 [151].

More recently, a splice variant of sst5, hsst5TMD4, forming a truncated receptor, was identified in several human pituitary tumors and, importantly, in GH-secreting tumors in which its expression was negatively correlated to clinical responsiveness to octreotide [7, 152].

In GH secreting adenomas, octreotide and lanreotide, mainly acting on sst2 and slightly less effectively on sst5, inhibit GH secretion [153], normalize IGF-1 serum levels, and cause tumor size reduction [154]. These analogs are the standard therapy for acromegalic patients, however the functional interactions between sst2 and 5 provide the rationale for studies using pasireotide (displaying a binding affinity sst5>sst2) [73].

Interestingly, the direct antiproliferative effects of SST analogs in pituitary adenoma cells are dissociated from the antisecretory effects, as shown by the differential responses to selective analogs [155, 156]. Inhibition of pituitary adenoma cell proliferation in response to native SST or lanreotide is mediated by increased PTP activity, as observed in primary cell cultures of human GH-secreting or nonfunctioning pituitary adenomas [157, 158]. Moreover, octreotide activation of SHP1 leading to tumor cell growth arrest through the regulation of the tumor suppressor Zac1, has been described in GH3 pituitary adenoma cells [110]. Finally, sst2 activation may also lead to caspase-mediated proapoptotic effects in somatotroph tumor cells treated with octreotide, via a PTP-dependent pathway [136], although the specific enzyme was not identified.

Prolactin-secreting adenomas which mainly express sst1 and sst5, often associated with D2R, show low sensitivity to somatostatinergic treatment that scarcely reduces prolactin secretion [149]. A study on primary prolactinoma cells overexpressing sst2 showed that the chimeric compound BIM-23A760 does not improve prolactin release suppression as compared to cabergoline, suggesting the predominance of dopaminergic signaling in prolactin release [159]. Thus, dopamine receptor agonists remain the most effective medical therapy for these tumors; however, a low percentage of patients did not respond to these drugs, probably because an overexpression of sst2, could represent a target for SST analogs.

ACTH-secreting tumor markedly express sst5 and D2R, and to a lesser extent sst1, 2 and 3 [160], but their pathobiology is more complex since, beside the corticotroph tumor, 70% of patients bear Cushing's disease showing circulating cortisol excess that can downregulate ACTH-cell sst2 expression leading to the lack of response to sst2 selective drugs, octreotide and lanreotide [74]. Pasireotide, acting on sst5, the most expressed receptor involved in ACTH secretion, may overcome the dependence of sst2 expression from high cortisol levels [161]. In fact, *in vitro *studies [162] showed that pasireotide significantly inhibited ACTH secretion from primary cultures of human ACTH-secreting pituitary adenomas, while clinical studies have shown that pasireotide can reduce urinary free cortisol levels in patients with Cushing's disease [163]. In this light, pasireotide was recently approved for the medical treatment of ACTH-secreting pituitary adenomas [76].

TSH-secreting tumors are extremely rare adenomas, highly expressing sst1, 2 and 5, in which sst2 plays a key role in the control of TSH secretion. Pharmacological therapy for TSH-secreting pituitary adenomas with sst2-preferring analogs, octreotide and lanreotide, induces normalization of hormone levels in the great majority of patients and tumor shrinkage in almost half of them [164]. However, besides sst2 expression the ratio sst5/sst2 and combined sst and D2R targeting was recently demonstrated to have a predictive value to long-term treatment with SST analogs and improve the response rate in octreotide-resistant tumors [165, 166].

GEP-NETs display high expression of SST receptors (higher for sst2 and sst5, lower for sst1, 3, and 4) [167], and thus SST analogs are potentially useful for the diagnosis and therapy of these tumors [44, 168]. A prospective study on the effect of octreotide LAR showed its efficacy in prolonging median time to tumor progression and increase the percentage of stable disease in patients with metastatic midgut NETs [169]. Currently, trials with lanreotide *versus* placebo in nonfunctioning pancreatic NET patients and pasireotide long-acting release in patients with metastatic NET are ongoing. Moreover, a promising therapeutic strategy recently proposed is the combination of octreotide or pasireotide and everolimus, an inhibitor of the protein kinase mammalian target of rapamycin (mTOR) controlling cell proliferation and survival [170]. Similar data were also reported in primary cultures of human nonfunctioning pituitary adenomas, in which the treatment with octreotide highly sensitized the cells to another mTOR inhibitor, rapamycin [116], thus supporting the possibility to increase SST efficacy by combining SST receptor agonists with other signal transduction inhibitors.

## 8. Somatostatin and Somatostatin Receptors in Non-Endocrine Tumors

Cytotoxic drugs still represent the main pharmacological approach for treatment of solid cancer although the induction of drug resistance, high toxicity, and poor selectivity often prevent successful outcome. However, novel promising therapeutic strategies such as targeted and biological therapy are aimed to reduce toxicity and improve selectivity. Among receptors for many regulatory peptides, SST receptors expressed on the membrane of tumor cells have provided the rationale for the development of SST agonists able to selectively target tumor cells. In fact, beside SST analogs, as previously described, cytotoxic SST derivatives (cytotoxic molecules conjugated to SST or analog backbone), and radiolabeled SST analogs for intracellular radiotherapy have been developed [171]. Some of these compounds are currently in preclinical and clinical trials, other have already reached a well-established clinical application. Here a concise view is given of SST receptor expression and SST analogs tested and used as antineoplastic agents, to the intensive research which is conducted in this field.

### 8.1. Prostate Cancer

All of the five SST receptors are detectable in prostate cancer tissues, with sst1 the most expressed, followed by sst5 and sst2 [172, 173]. Many studies using SST analogs (octreotide, lanreotide, pasireotide) have been performed in prostate cancer cell lines [174, 175] highlighting both antiproliferative and proapoptotic effects. In LNCaP cells, an interaction between SST receptors and D2R was also documented. These cells constitutively express sst1, 2 and 5 and D2R, and their activation by receptor selective SST agonists or SST/dopamine chimeras, that synergistically activates sst2/D2R dimers, significantly inhibited cell proliferation [55]. Similarly, the use of SST analogs conjugated with doxorubicin (AN-162) showed a powerful *in vitro* and *in vivo* efficacy in experimental prostate cancer model, being also able to affect metastasization [176]. However, clinical studies using octreotide and lanreotide as single agents for the treatment of prostate cancer patients did not show significant results [177], while combination therapies, including octreotide LAR plus dexamethasone and the bisphosphonate zoledronic acid produced objective responses and symptomatic improvement in androgen ablation-refractory patients [178, 179].

### 8.2. Breast Cancer

Breast tumors express all SST receptors at high level, sst2 being the predominant subtype [180]. Experimental studies support the efficacy of octreotide and lanreotide in the control of tumor growth *in vitro* and in xenograft models [181–183]. Recently, the truncated sst5 variant, sst5TMD4, related to the abnormal responses to SST analogs in pituitary tumors has been identified in poorly differentiated human breast cancers, where it correlates and interacts with sst2 altering its signaling and affecting tumor pathophysiology, while it is absent in normal mammary gland [184]. 

IGF-1 levels are associated with breast hyperplasia and cancer risk [185]. IGF-1 effects might be blocked by SST analogs though an indirect regulation mediated by the inhibition of GH release. *In vivo* studies reported that pasireotide could affect IGF-1 activity as far as cell division and inhibition of apoptosis, but coadministration of tamoxifen did not result in greater effects [186, 187]. In a rat model of breast hyperplasia [188] this effect was prevented by pasireotide and tamoxifen coadministration, while octreotide was less effective, while in another study the SST analog shows the same activity as tamoxifen in preventing GH- and estrogen-induced mammary hyperplasia [188].

Controversial results were obtained in breast cancer patients treated with SST analogs. In a phase II study, lanreotide + tamoxifen induced a 50% of overall objective response rate, with a 12.5% of complete responses, and similar responses were observed using the octreotide/tamoxifen association [189, 190]. However, while a subsequent meta-analysis seemed to confirm these results when SST analogs were given as first-line therapy [191], several phase III studies did not show any clinical improvement by the addition of octreotide LAR to tamoxifen [192–194], even in cases in which changes in circulating IGF-1 and C-peptide levels were statistically significant [195]. 

### 8.3. Cancers of the Gastrointestinal Tract

Cancers of the digestive tract differently express SST receptors according to the localization [196]. In particular, colorectal malignancies predominantly express sst1 (65%) followed by sst5 (39%) and sst2 (36%) [197], hepatocellular carcinomas express mainly sst5, although sst1, 2, and 3 are also often detected (about 40–60%) [198], while in gastric carcinomas sst2 and 5 are very commonly expressed, although sst3 is detected in several cases [199]. Moreover, also gastrointestinal stromal tumors (GIST) were reported to express all five SST receptors in variable percentages [200, 201].

Antiproliferative activity of SST and analogs mediated by sst3 and 5 was demonstrated *in vitro *in different colon cancer cell lines through a PTP-dependent inhibition of COX2 expression and activity [202]. When translated to the clinical studies in patients with advanced colorectal cancer controversial results were obtained. In a randomized study in 46 nonresponder patients to conventional chemotherapy the administration of octreotide significantly increased overall survival in comparison with patients receiving supportive care (24 *versus* 12 weeks), but no different effects than placebo were observed in a larger phase III trial in asymptomatic colon cancer patients [203, 204].

As far as hepatocellular cancer, proliferation was reduced by octreotide in human cell lines, [205] and by pasireotide that synergized with celecoxib to induce apoptosis *in vitro* and to prolong the survival of nude mice bearing hepatocarcinoma xenografts [206]. 

A phase II clinical trial using octreotide LAR in combination with sorafenib showed partial responses or disease stability in about 75% of the patients [207], but these results were not confirmed in a phase III trial, in which octreotide LAR was compared to placebo [208]. 

Hypermethylation of SST promoter was identified as potential mechanism of gastric cancer development [209], clearly highlighting the potential role for SST receptors in gastric cell proliferation. Octreotide reduced gastric carcinoma cell line proliferation *in vitro* interfering with Akt and telomerase activity [210], an effect that was dependent on the expression of sst3. The COX2 inhibitor rofecoxib, administered with octreotide in a gastric cancer xenograft model, caused an almost total tumor growth suppression due to induction of apoptosis [211]. These *in vitro* results were confirmed in clinical studies in which octreotide potentiated celecoxib effect in gastric carcinoma patients, increasing both necrosis and apoptosis and inhibiting angiogenesis [212]. Antivasculogenesis effects of SST gastric cancer patients were ascribed to a direct downregulation of both VEGF and its receptor VEGFR3 activity induced by the peptide in an open-label, randomized trial including 60 patients [213].

Above evidence suggests that SST and its analogs can be potentially useful as adjuvant therapy to improve the outcome of some neoplasms of the digestive tracts, although the large discrepancies between experimental and clinical studies clearly suggest that the exact comprehension of the mechanisms responsible of the antiproliferative effects mediated by SST receptors will require further studies.

### 8.4. Brain Tumors

Glioblastoma multiforme (GBM), the most aggressive primary brain cancer, expresses multiple SST receptors [214], being sst1 and sst2 the most frequent in both glioma tissues and cell lines [133, 215]. *In vitro*, the activation of SST receptors leads to antiproliferative and anti-invasive effects [133, 216]. Besides the expression of SST receptors, in both GBM cell lines and primary cell cultures specific signaling pathways regulated upon SST receptors activation were identified as determinants of the transduction of the growth-inhibitory effects of SST, showing a prominent role for the activation of PTP*η* [133, 134]. Subtype-selective agonists for each of SST receptors provide a direct approach to define individual role of SST receptors in the antiproliferative effects of SST [69]. Using C6 rat glioma cells, an experimental model that *in vivo* closely reflects invasion and neovascularization of human GBM growth [217], the efficacy of SST analogs was assayed on tumor growth in nude mice [70]. Single agonists of sst1, 2, and 5 affected both tumor growth and neoangiogenesis via the inactivation of ERK1/2 and the upregulation of p27^Kip1^, representing a common intracellular pathway for all the receptors. Interestingly, while the sst5 agonist (BIM-23206) was maximally effective on tumor development, sst1 and sst2 selective agonists (BIM-23745 and BIM-23210) were more efficacious on tumor vascularization [70]. Thus, it was hypothesized that the simultaneous activation of different SST receptor subtypes will improve the potential of this antitumor therapeutic approach [218].

Besides GBM, another common primary intracranial neoplasia is meningioma that despite a general low aggressive clinical behavior, includes, in few cases, high-grade lesions that recur after surgery and radiotherapy. The high incidence of SST receptors in human meningiomas is known since decades [219] and the inhibitory role of SST in the control of proliferation in primary cultures of human meningioma cells has been described [220]. The rationale for the use of SST analogs in the control of intracranial meningioma is supported by the widespread expression of SST receptors in this tumor and by the fact that currently no adjuvant therapy for meningioma is available in patients with unresectable or radioresistant lesions [221]. Limited clinical data are currently available about SST analogs *in vivo*, but SST antiangiogenic activity might be useful in refractory meningiomas as reported by The Central Nervous System National Comprehensive Cancer Network guidelines that suggests as treatment options hydroxyurea, interferon-*α*, or octreotide LAR [222]. In addition, a phase II trial for recurrent or progressive patients treated with pasireotide is ongoing.

The antitumor effects described above are to be considered as examples and not exhaustive of all tumors expressing SST receptors in which preclinical and clinical investigations have been performed with SST analogs. However, the main relevant results reviewed can highlight the intriguing option to add SST analogs to cytotoxic or targeted drugs to improve the clinical outcome of various endocrine and non-neuroendocrine neoplasms. 

## 9. Conclusions

SST receptors represent a potential relevant drug target for the treatment of several tumor histotypes. In fact, starting from the identification of the expression, and in some cases overexpression, of these receptors in several human tumors, several clinical trials were performed to assess the possible clinical efficacy of SST agonists in almost all tumor types. However, to date contrasting or clearly negative results were obtained, with the only significant exceptions of pituitary adenomas and, possibly, GEP-NETs. In fact, in these tumors, a significant inhibition of hormone secretion (responsible of most clinical symptoms) and, although less evident in all the tumors, cell cycle arrest was documented. However, in recent years a significant advancement in understanding the biology of SST receptors (mainly the mechanisms of dimerization and cellular trafficking) and the identification of PTPs as main mediators of the antiproliferative effects may help in the comprehension for the reason in the so far disappointing results for most of the tumor treated. In particular, data on the balance of SST receptor subtype expression [166] and the expression of specific PTPs [129] may help in the identification of subset of tumors most likely to be responsive to SST analogs treatment, thus providing a step ahead in the personalized treatment of patients in oncology. Finally, the recent development of SST-based PRRT could represent an adjunctive approach to treat in other ways non-responder tumors.

## Figures and Tables

**Table 1:** Binding affinities of native SST and synthetic agonists for SST receptor subtypes.

Ligands		Binding affinity (IC_50 _nM)				
		sst1	sst2	sst3	sst4	sst5
Endogenous	SST-14	**0.1–2.26**	**0.2–1.3**	**0.3–1.6**	**0.3–1.8**	**0.2–0.9**
	SST-28	**0.1–2.2**	**0.2–4.1**	**0.3–6.1**	**0.3–7.2**	**0.05–0.4**
	CST-14	**2.1**	**0.5**	**3.8**	**18.2**	**0.9**
	CST-17	**0.25–7.0**	**0.6–0.9**	**0.4–0.6**	**0.5–0.6**	**0.3–0.4**

Synthetic peptides in clinical use	Octreotide	>1000	**0.4–2.1**	4.4–34.5	>1000	**5.6–32**
	Lanreotide	>1000	**0.5–1.8**	43–107	>1000	**0.6–14**
	Pasireotide	**9.3**	**1**	**1.5**	>100	**0.16**

Synthetic peptides in clinical trials	Vapreotide	>1000	**0.2–5.4**	31	45	**0.7**
	Somatoprim	>1000	**3**	>100	**7**	**6**

Synthetic peptides in experimental use	Seglitide	>1000	**0.1–1.5**	27–36	>1000	**2–23**
	BIM-23268	18.4	15.1	61.6	16.3	**0.37**
	BIM-23745	**42**	>1000	>1000	>1000	>1000
	BIM-23926	**4**	>1000	>1000	>1000	>1000
	BIM-23120	>1000	**0.34**	412	>1000	213,5
	BIM-23206	>1000	166	>1000	>1000	**2.4**
	BIM-23704	6.3	**1.4**	43.2	>1000	115
	BIM-23190	>1000	**0.35**	215	>1000	11.2
	BIM-23A799	**2.5**	**0.3**	**0.6**	>1000	**0.6**
	KE108	**2.6**	**0.9**	**1.5**	**1.6**	**0.65**

Nonpeptide agonists	L-797,591	**1.4**	1875	2240	170	>1000
	L-779,976	>1000	**0.05**	729	310	>1000
	L-796,778	>1000	>1000	**24**	>1000	>1000
	L-803,087	199	>1000	1280	**0.7**	>1000
	L-817,818	**3.3**	52	64	82	**0.4**

Chimeric SST/DA compounds	BIM-23A757^a^	ND	**0.58**	ND	ND	104.4
	BIM-23760^b^	622	**0.03**	160	>1000	42
	BIM-23A761^c^	ND	**0.01**	ND	ND	**3.7**

Antagonists	ODN-8	>10000	>10000	**6.7**	>10000	>10000
	BN81658	>1000	>1000	**1.58**	>1000	>1000
	Cyn154806	>1000	**3.6**	150	650	**20**

High affinity for individual SST receptors is reported in bold.

D2R IC_50_: ^a^7.9, ^b^15, ^c^27 nM, ND: not determined.

**Table 2:** Main signalling systems regulated by the activation of SST receptors.

	sst1	sst2	sst3	sst4	sst5
cAMP production					
Adenylyl cyclase	*▼*	***▼***	***▼***	***▼***	***▼***
Tyrosine phosphatases					
PTP*η*	**▲**	**▲**	**▲**	**▲**	**▲**
SHP-1	∘	**▲**	**▲**	∘	**▲**
SHP-2	**▲**	**▲**	**▲**	**▲**	**▲**
Ion channels and transporters					
Ca^2+^ channels	***▼***	***▼***	∘	∘	***▼***
K^+^ currents	**▲**	**▲**	**▲**	**▲**	**▲**
Na^+^/H^+^ exchanger 1 (NHE1)	***▼***	***▼*/▲**	∘	**▲**	∘
MAPK					
ERK1/2	***▼*/▲**	***▼*/▲**	***▼***	**▲**	***▼***
p38	∘	**▲**	∘	**▲**	∘
JNK	∘	***▼***	∘	∘	**▲**
Tyrosine kinases					
c-Src	**▲**	**▲**	∘	∘	∘
JAK2	**▲**	**▲**	∘	∘	∘
Phospholipid kinases					
PI3K	***▼*/▲**	***▼*/▲**	∘	∘	∘
Cyclin-dependent kinase inhibitors					
p27^kip1^	**▲**	**▲**	∘	∘	∘
p21^Cip1^	**▲**	∘	∘	∘	∘
Zac1	∘	**▲**	∘	∘	∘
Nitric oxide synthases					
nNOS	∘	**▲/*▼***	***▼***	∘	***▼***
eNOS	***▼***	***▼***	***▼***	∘	∘
Apoptotic pathways					
p53	∘	∘	**▲**	∘	∘
BAX	∘	∘	**▲**	∘	∘
Bcl-2	∘	∘	***▼***	∘	∘

▲: activation, *▼*: inhibition, ∘: no effects.
